# Mimicking the Nitric Oxide‐Releasing and Glycocalyx Functions of Endothelium on Vascular Stent Surfaces

**DOI:** 10.1002/advs.202002330

**Published:** 2020-09-27

**Authors:** Nan Lyu, Zeyu Du, Hua Qiu, Peng Gao, Qin Yao, Kaiqin Xiong, Qiufen Tu, Xiangyang Li, Binghai Chen, Miao Wang, Guoqing Pan, Nan Huang, Zhilu Yang

**Affiliations:** ^1^ Key Lab of Advanced Technology of Materials of Education Ministry School of Materials Science and Engineering Southwest Jiaotong University Chengdu Sichuan 610031 China; ^2^ Department of Urology Affiliated Hospital of Jiangsu University 438 Jiefang Road Zhenjiang Jiangsu 212001 China; ^3^ Institute for Advanced Materials School of Materials Science and Engineering Jiangsu University 301 Xuefu Road Zhenjiang Jiangsu 212013 China

**Keywords:** cardiovascular stents, endothelium mimicking coatings, glycocalyx component, nitric oxide, surface bioengineering

## Abstract

Endothelium can secrete vasoactive mediators and produce specific extracellular matrix, which contribute jointly to the thromboresistance and regulation of vascular cell behaviors. From a bionic point of view, introducing endothelium‐like functions onto cardiovascular stents represents the most effective means to improve hemocompatibility and reduce late stent restenosis. However, current surface strategies for vascular stents still have limitations, like the lack of multifunctionality, especially the monotony in endothelial‐mimic functions. Herein, a layer‐by‐layer grafting strategy to create endothelium‐like dual‐functional surface on cardiovascular scaffolds is reported. Typically, a nitric oxide (NO, vasoactive mediator)‐generating compound and an endothelial polysaccharide matrix molecule hyaluronan (HA) are sequentially immobilized on allylamine‐plasma‐deposited stents through aqueous amidation. In this case, the stents could be well‐engineered with dual endothelial functions capable of remote and close‐range regulation of the vascular microenvironment. The synergy of NO and endothelial glycocalyx molecules leads to efficient antithrombosis, smooth muscle cell (SMC) inhibition, and perfect endothelial cell (EC)‐compatibility of the stents in vitro. Moreover, the dual‐functional stents show efficient antithrombogenesis ex vivo, rapid endothelialization, and long‐term prevention of restenosis in vivo. Therefore, this study will provide new solutions for not only multicomponent surface functionalization but also the bioengineering of endothelium‐mimic vascular scaffolds with improved clinical outcomes.

## Graphical abstract

An endothelium‐mimicking coating NO‐releasing property and grafted glycocalyx molecules on a cardiovascular stent is reported. The dual functions endow the stent with antithrombosis, smooth muscle cell (SMC) inhibition, and endothelial cell (EC)‐compatibility, which result in efficient antithrombogenesis ex vivo, rapid endothelialization, and long‐term prevention of restenosis in vivo.

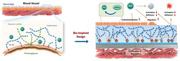

## 1 Introduction

Cardiovascular disease represents one of the most serious health problems in modern society with the highest rate of death and disability.^[^ ^1^ ^]^ It is usually associated with a build up of fatty deposits inside arteries (i.e., atherosclerosis) or pathological arterial thrombus formation, characterized by arterial obstruction. In clinic, interventional vascular stents are used to ensure the patency of blood vessels.^[^ ^2^ ^]^ However, endothelium injury caused by stent expansion will provoke thrombogenic and inflammatory reactions, which may induce sudden death or non‐fatal myocardial infarction, let alone the relieving of arterial obstruction.^[^ ^3^ ^]^ In addition, the damages of endothelium will also trigger neointimal proliferation during vascular reconstruction. Excessive smooth muscle cells (SMC) migration and proliferation (i.e., hyperplasia), together with thrombogenesis around the implant, most likely lead to a typical complication, that is, the in‐stent restenosis (ISR).^[^ ^4^ ^]^ Although the immediate reason of thrombosis and hyperplasia is stenting‐caused damages and dysfunctions of endothelium around the implantation region, a critical intrinsic factor that cannot be ignored is the bioinertness and lack of endothelial functions (e.g., the hemocompatibility and the ability to regulate vascular cell behaviors)^[^ ^5^ ^]^ on the surfaces of implanted stents. From the bionic point of view, an ideal vascular stent should be surface endowed with endothelial functions, which will facilitate an immediate inhibitory effect on thrombogenesis and hyperplasia after stent implantation.

In circulations, endothelium plays a very important role in maintaining the vascular homeostasis.^[^ ^6^ ^]^ Endothelial cells (ECs) can secrete vasoactive mediators and produce specific extracellular matrix (ECM) molecules, which contribute greatly to the excellent thromboresistance of endothelium and its positive functions for vascular wall remodeling.^[^ ^7^ ^]^ As the typical vasoactive mediator and ECM molecule, NO (nitric oxide, gaseous signaling molecule secreted by ECs)^[^ ^8^ ^]^ and glycocalyx (polysaccharide matrix on ECs)^[^ ^9^ ^]^ provide a good illustration of the endothelial functions for both remote and close‐range regulation of vascular microenvironment. Continuous and stable NO release into the vascular microenvironment has a significant inhibitory effect on platelet activation, thrombus formation, SMC migration and proliferation as well as a positive healing effect on atherosclerotic lesions^[^ ^10^ ^]^; and endothelial glycocalyx can directly modulate the ECs‐blood interactions to inhibit the adhesion of platelets, leukocytes, erythrocytes, and also the plasma macromolecular substances.^[^ ^11^ ^]^ Therefore, vascular stents with NO‐releasing property and surface engineered endothelial glycocalyx molecules may provide highly biomimetic endothelial functions for the prevention of thrombosis, hyperplasia and restenosis.

To this end, surface bioengineering on the implants is indispensable.^[^ ^12^ ^]^ Intuitively, molecular modification of vascular stent surfaces with both NO‐generating compounds and glycocalyx components is one of the most straightforward ways to mimic the complex functions of endothelium. Currently, surface modification of vascular stents with monocomponent bioactivity is easy of handling. For example, the key components of glycocalyx like hyaluronan or heparin can be efficient grafted on vascular stents for inhibition of endothelium‐blood interactions^[^ ^13^ ^]^; and the glutathione peroxidase (GPx)‐like transition metal ion Cu^2+^,^[^ ^14^ ^]^ chelating with a cyclen DOTA (1,4,7,10‐tetraazacyclododecane‐*N*,*N*′,*N*″,*N*″′‐tetraacetic acid), can be easily immobilized on stent surfaces for NO‐generation via catalytical decomposing of endogenous or synthetic S‐nitrosothiols (RSNOs). However, the combination of NO‐releasing and endothelial glycocalyx molecules has rarely been explored, despite the higher endothelium‐mimicking nature of such kind of dual‐functional surfaces compared to those of monocomponent ones. This is probably due to the disadvantages of traditional surface modification methods for multiple components, for example, the poor controllability, operability and reproducibility.^[^ ^15^ ^]^ In this context, improved surface bioengineering strategies capable of easy‐to‐handle and controllable multicomponent modification is highly desired, in particular, for simultaneous mimicking of the NO‐releasing and endothelial glycocalyx functions on vascular stent surfaces.

**Figure 1:**
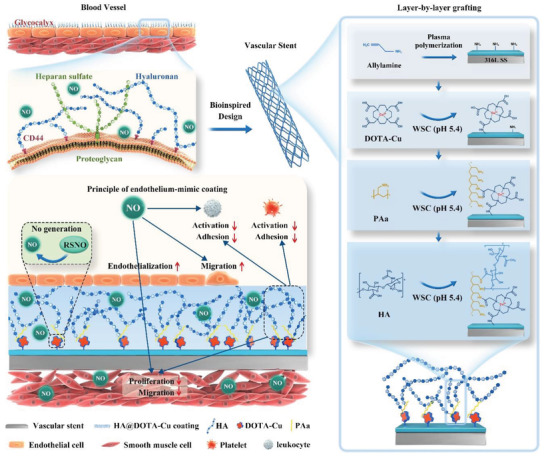
Endothelium‐mimic dual‐functional coating on vascular is achieved by layer‐by‐layer grafting NO‐generating species and the glycoside component hyaluronan. The anticoagulant hyaluronan surface and NO release can synergistically inhibit platelet activation and smooth muscle proliferation on the vascular stent, leading to the improved properties for prevention of thrombosis and restenosis.

Herein, we report a layer‐by‐layer grafting strategy to create a biomimetic surface with endothelium‐like dual functions on the cardiovascular scaffolds (**Figure** **1**). In this study, the NO‐generating Cu^2+^‐chelated DOTA and the typical glycocalyx component HA were sequentially immobilized on plasma polymeric allylamine (PPAm)‐coated stents^[^ ^16^ ^]^ through water‐phase amidation. The layer‐by‐layer grafting strategy enables independent control of the biomolecular density and content on each grafting layer, thus facilitating the adjusting and optimizing surface functions in multicomponent modifications. Specifically, the carboxyl groups of DOTA one side were first used to conjugate with the amino groups of PPAm on stents, leaving the residue carboxyl groups for a second grafting. To get high HA grafting density, polyallylamine (PAa) was further employed to amplify the reaction sites by transferring the carboxyl groups into multiple amino groups. HA was then amidated on the surface, finally obtaining the layer‐by‐layer grafted vascular stents with NO‐releasing and endothelial glycocalyx functions. In this case, the dual‐functional surfaces might posses typical endothelium‐like properties, such as NO releasing, antiplatelet and vascular‐cell‐preferred surface properties.^[^ ^15^, ^17^ ^]^ In addition, the synergistic effects of NO‐releasing and endothelial glycocalyx functions in vivo would also lead to perfect antithrombogenesis,^[^ ^18^ ^]^ rapid endothelialization, and finally, the prevention of restenosis. In a word, the positive results presented in this work will provide new solutions for not only multicomponent surface modification but also the fabrication of endothelium‐mimic vascular scaffolds with improved clinical outcomes.

## 2 Results and Discussion

### 2.1 Fabrication of Endothelium‐Mimic Dual‐Functional Coating

**Figure 2:**
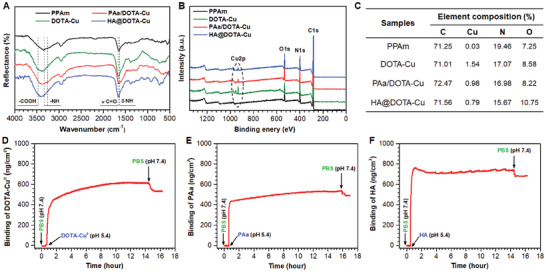
A) GATR‐FTIR spectra, B) XPS wide‐scan survey, and C) the corresponding elemental compositions on the surfaces of PPAm, DOTA‐Cu, PAa/DOTA‐Cu, and HA@DOTA‐Cu. D,E) Real‐time QCM‐D analysis on the grafting process of DOTA‐C^2+^(D), PAa (E) and HA (F). Data are presented as mean ± SD (*n* = 4).

To facilitate molecular conjugations, allylamine (Aa) was used for surface amination via capacitive plasma deposition. Since 316L stainless steel (SS) represents one of the widely used materials for vascular stents, the mirror‐polished 316L SS substrates were employed in this work. The aminated (PPAm‐deposited) 316L SS substrates were sequentially conjugated with DOTA‐Cu^2+^, PAa and HA. The modified samples at each step of molecular conjugations were denoted as PPAm, DOTA‐Cu, PAa/DOAT‐Cu, and HA@DOTA‐Cu, respectively. After each step of molecular conjugations, the surface chemical components and structures were characterized by Grazing incidence attenuated total reflection Fourier transform infrared spectroscopy (GATR‐FTIR) and X‐ray photoelectron spectroscopy (XPS, K‐Alpha, Thermo Electron, USA). As shown in **Figure** **2**A, the absorption bands of DOTA‐Cu and HA@DOTA‐Cu at 3600–3100 cm^−1^ were significantly broadened due to the introduction of −COOH stretching vibration peak. In addition, the significant enhanced peaks of C = O stretching vibration (1680 cm^−1^) and N‐H deformation vibration (1635 cm^−1^) in the spectra of DOTA‐Cu, PAa/DOTA‐Cu, and HA@DOTA‐Cu indicated the success of condensation reaction between ‐COOH and ‐NH_2_.

XPS analysis also showed significant differences of chemical composition between the groups of DOTA‐Cu, PAa/DOTA‐Cu, and HA@DOTA‐Cu (Figure 2B,C). Upon the conjugation of DOTA‐Cu^2+^, the copper element appeared, and only a slight decrease could be observed after further step immobilization (i.e., in the groups of PAa/DOAT‐Cu and HA@DOTA‐Cu). This indicated successful introduction of DOTA‐Cu^2+^ on aminated substrates, and further, the immobilized Cu^2+^ could tolerated subsequent molecular conjugations (Figure S1, Supporting Information). The significant increase of O1s signal in the groups of DOTA‐Cu and HA@DOTA‐Cu after conjugation also implied the success of DOTA and HA immobilizations. In order to further understand the chemical composition, the C1s peaks of XPS were applied with peak fitting procedures (Figure S2, Supporting Information). The appearance of peak at 288.2 eV in the C1s high‐resolution spectrum suggested the conjugation of DOAT‐Cu^2+^ on the aminated substrates. A visible increase or decrease of shoulders at 286 and 286.8 eV further indicated the condensation reactions of ‐NH_2_ with ‐COOH. Taken together, these results preliminarily demonstrated the feasibility of sequentially amination for fabrication of dual‐functional surface.

The grafting density of functional molecules is an important index for efficient biological effects. Real time Quartz crystal microbalance equipped with dissipation (QCM‐D, Q‐sense AB, Sweden) analysis on the changes of grafted molecular mass was then performed. As shows in Figure 2D, we found the compound DOTA‐Cu^2+^ could be efficiently immobilized on the substrate within 1 h. Even after 2 h of PBS elution, 550 ng cm^−2^ DOTA‐Cu^2+^ chelate could stay on the surface. At the step of PAa grafting, a fast reaction between the grafted DOTA and PAa could be observed (Figure 2E). After 10 min, approximately 480 ng cm^−2^ PAa was immobilized on the DOTA‐Cu surface. Under the same reaction conditions, HA could also be immobilized on the surface of PAa/DOTA‐Cu within 1 h, resulting in significant increased surface wettability (Figure S3, Supporting Information). QCM‐D analysis also revealed that 680 ng cm^−2^ of HA could be grafted (Figure 2F). These results indicated that the sequentially layer‐by‐layer grafting strategy could be used to obtain dual‐functional surfaces with considerable grafting density.

### 2.2 Endothelium‐Mimic NO‐Releasing Property

**Figure 3:**
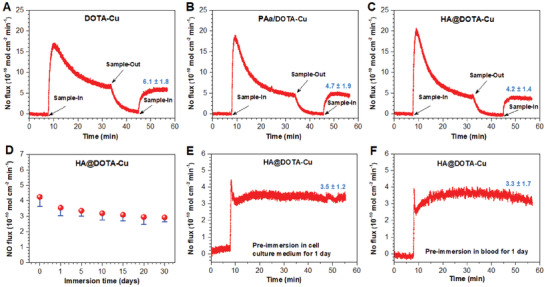
A–C) Real‐time NO flux from DOTA‐Cu, PAa/DOTA‐Cu, HA@DOTA‐Cu after immersion in PBS supplemented with NO donor (10 µm GSNO and 10 µm GSH). D) Time‐dependent NO flux of HA@DOTA‐Cu. E,F) NO flux of HA@DOTA‐Cu after preimmersion in cell culture medium and blood for 1 day.

With the DOTA‐Cu^2+^‐decorated surfaces in hand, the NO catalytic release ability was then evaluated using a chemiluminescence NO analyzer. As expected, the samples with chelated Cu^2+^ all showed efficient NO‐releasing property in PBS solution supplemented with 10 µm reducing agent l‐glutathione reduced (GSH)and 10 µm GSNO (it is an endogenous NO donor) (**Figure** **3**A‐C). Thereinto, the DOTA‐Cu surface had the highest NO flux up to 6.1±1.8 × 10^−10^ mol cm^−2^ min^−1^. In contrast, the PAa/DOTA‐Cu and HA@DOTA‐Cu surfaces showed slightly lower NO flux (4.5±1.9 × 10^−10^ and 4.0±1.4 × 10^−10^ mol cm^−2^ min^−1^, respectively), probably due to the shielding effect of subsequent molecular grafting. Since the stability of NO catalytic release is closely related to long‐term use of stents, ageing studies were performed for HA@DOTA‐Cu by soaking the samples in PBS for 1–30 days, and then their NO release stability was evaluated every five days. The results showed that the NO catalytic release ability showed a slight decrease in the first 10 days, but it can stabilize around 3.0±0.3 × 10^−10^ mol cm^−2^ min^−1^ for more than 2 weeks (Figure 3D), indicating the suitability for long‐term use. To further verify that the applicability of the NO‐release surfaces in blood or tissue fluid, the NO flux after immersing with cell culture medium and blood were tested. The NO catalytically released ability only showed negligible decreases with stable flux around 3.5 ± 1.2 × 10^−10^ and 3.3 ± 1.7 × 10^−10^ mol cm^−2^ min^−1^, respectively (Figure 3E,F). These results jointly demonstrated the feasibility of these DOTA‐Cu^2+^‐chelated surfaces for long‐term use in vivo.

### 2.3 In Vitro Antiplatelet Property

**Figure 4:**
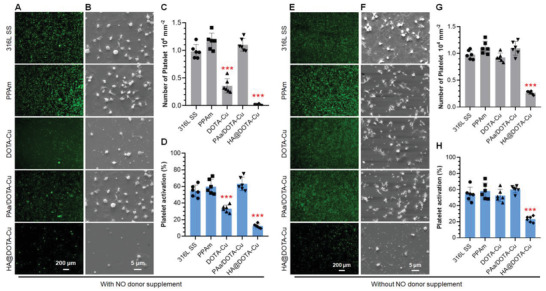
A,E) Fluorescence and B,F) SEM images of platelet adhesion on 316L SS surface, PPAm, DOTA‐Cu, PAa/DOTA‐Cu, and HA@DOTA‐Cu surfaces with or without NO donor (10 µm GSNO and 10 µm GSH). The amount of C,G) adherent platelets and D,H) activated platelets were obtained by counting and the GMP‐140 assay. Data presented as mean ± SD and analyzed using a one‐way ANOVA, ::*p* < 0.01, :::*p* < 0.001.

At the early stage of post‐implantation, thrombogenesis on stents are the crucial problems confronted. Therefore, anticoagulation and prevention of thrombogenesis is one of the most essential requirements for all blood contact materials, especially the cardiovascular stents. Since thrombogenesis involves a series of biochemical processes like platelet aggregation, coagulation, and fibrinolysis, we checked the in vitro antiplatelet property first. As shown in **Figure** **4**A‐D, the monofunctional group DOTA‐Cu and dual‐functional group HA@DOTA‐Cu both showed significant inhibition of platelet adhesion and activation with the NO donor supplement. In contrast, the controls including the bare 316L SS and PPAm substrates showed almost no inhibition in the amount and activation rates of adherent platelets. We also found that, without donor supplement, only the HA@DOTA‐Cu group could significantly inhibit the platelet adhesion and activation (Figure 4E‐H). Nevertheless, the dual‐functional HA@DOTA‐Cu in NO donor supplemented medium exhibited the most potent antiplatelet property. This result implied the importance of NO and glycocalyx molecules for the synergetic inhibition of thrombogenesis in vascular microenvironment. The dual‐functional HA@DOTA‐Cu surface thus may show perfect endothelium‐like antithrombogenesis in vivo.

### 2.4 Ex Vivo Antithrombogenic Propertie*s*

**Figure 5:**
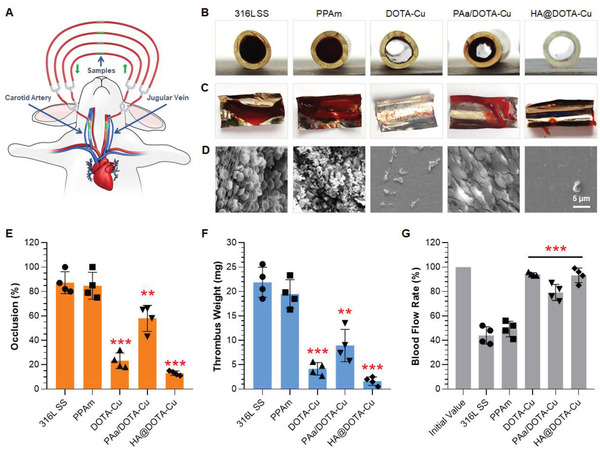
A) The ex vivo circulation thrombogenicity model of rabbit. B) Cross‐sectional photographs of tubing containing different foils exposed to blood flow supplemented with NO donor for 2 h in a rapid AV shunt model without external heparin. C) Photographs of thrombus on the foils. D) SEM images of the adhered platelets and fibrinogen. Quantitative results of E) occlusion rates, F) the thrombus weights, and G) blood flows in different groups. Data presented as mean ± SD (*n* = 4) and analyzed using a one‐way ANOVA, :*p* < 0.05, ::*p* < 0.01, :::*p* < 0.001.

To verify the practical antithrombogenic efficacy, an ex vivo catheter blood extracorporeal circuits experiment was further performed. 316L SS foils with different surface treatments were curled up and placed onto a commercially available medical catheter and then connected to in a rabbit arteriovenous shunt circuit (**Figure** **5**A).

After 2 h of ex vivo blood circulation with the supplement of NO donor, all the foils were collected, and the permeability, thrombus weights and blood flow rates in the circuit were evaluated. As the photos shown in Figure 5B,C, the NO‐releasing groups all showed better permeability and less thrombus than the other controls. Scanning electron microscopy (SEM) further confirmed this result that only a small amount of cruor was observed on the groups of DOTA‐Cu and HA@DOTA‐Cu (Figure 5D). Quantitative analysis revealed that the occlusion rates in groups of bare 316L SS, PPAm, DOTA‐Cu, PAa/DOTA‐Cu, and HA@DOTA‐Cu were 87.3%, 84.8%, 23.3%, 58.0%, and 12.0%, respectively (Figure 5E). Correspondingly, the thrombus weights and blood flow rates showed a consistent result (Figure 5F,G), that is, the dual‐functional HA@DOTA‐Cu group exhibited significantly reduce in thrombogenesis and improved patency than the others. The result was also in line with the antiplatelet properties in vitro, which jointly demonstrated the importance of endothelium‐mimic NO‐releasing and glycocalyx functions for the synergetic inhibition of thrombogenesis in a vascular microenvironment.

### 2.5 In Vitro Vascular Cell Growth Behaviors

**Figure 6:**
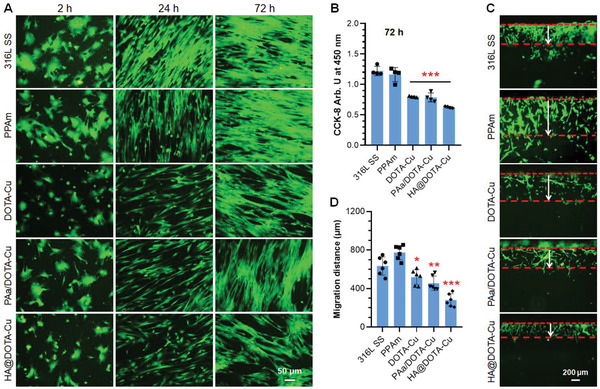
A) Fluorescence staining of human umbilical artery smooth muscle cells (HUASMCs) on different surfaces after culture for 2, 24 and 72 h with NO donor supplement (10 µm GSNO and 10 µm GSH). B) The proliferation of HUASMCs with NO donor for 72 h. C,D) Migration of HUASMCs on different surfaces after 1 day of culture with donor supplement. Arrows show the distance of cell migration. Data are presented as the mean ± SD (*n* = 4) and analyzed using one‐way ANOVA, :*p* < 0.05, ::*p* < 0.01, :::*p* < 0.001.

In addition to the antiplatelet and antithrombogenic ability, the effect of dual‐functional surfaces on (SMC inhibition and EC promotion is also important because of the correlation to neointimal hyperplasia and endothelial regeneration.^[^ ^19^ ^]^ Usually, stent implantation causes endothelium damage, which inevitably leads to the leak of SMCs. Under the condition without the control of ECs, excessive SMCs growth and delayed re‐endothelialization on the surface of stents will result in hyperplasia and in‐stent restenosis.^[^ ^17^, ^20^ ^]^ Thus, in this section, we evaluated the growth and migration behaviors of vascular cells on different surfaces. The SMCs behaviors on different substrates were first investigated. As shown in **Figure** **6**A,B, all the NO‐releasing surfaces (i.e., the Cu^2+^‐chelated surfaces with NO donor supplement) showed remarkable inhibitory effect on the adhesion and proliferation of SMCs. In contrast, all groups showed no significant inhibition on SMCs without donor supplement (Figure S4, Supporting Information). The results confirmed that the NO‐releasing property is crucial for excessive SMCs growth and hyperplasia prevention. It is worth mentioning that, although the monofunctional DOTA‐Cu exhibited the most potent NO‐releasing ability, its inhibitory effect on SMCs still was weaker than the group of HA@DOTA‐Cu with both NO‐releasing and glycocalyx functions. This finding preliminarily confirmed our hypothesis that the dual‐functional endothelium‐mimic surface would provide the better vascular‐stent‐preferred properties.

Previous studies also indicated that the NO‐caused inhibition of SMCs also reflected in their migration behavior. This is desired for vascular stents since the inhibition of SMCs migration on the stents could effectively reduce the probability of restenosis after stent implantation. To confirm the deduction, the migration distances of SMCs on different substrates was record after 1‐day culture. In the absence of NO donor in medium, the migration of SMCs on all surfaces showed no significant difference (Figure S5, Supporting Information). Upon the addition of NO donor, the properties of cell migration on the NO‐releasing surfaces (i.e., DOTA‐Cu, PAa/DOTA‐Cu, and HA@DOTA‐Cu) changed remarkably (Figure 6C,D). Amongst, the SMCs migration distance on the dual‐functional (HA@DOTA‐Cu) surfaces showed the maximal decrease of 55.8% compared the bare 316L SS surface. This result, together with the inhibition of SMCs adhesion and proliferation as well as the antithrombogenesis, further demonstrated the importance of NO and glycocalyx molecules for vascular stents.

**Figure 7:**
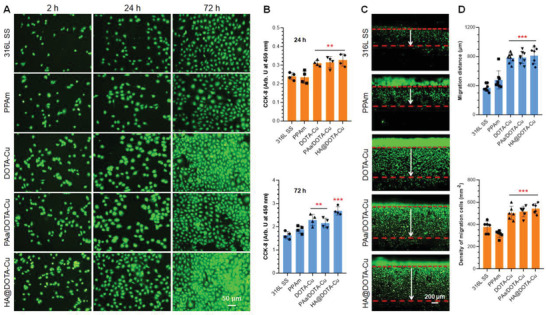
A) Fluorescence staining of human umbilical vein endothelial cells (HUVECs) on different surfaces after culture for 2, 24 and 72 h with NO donor (10 µm GSNO and 10 µm GSH). B) The proliferation of HUVECs in culture media with donor for 24 and 72 h. C,D) Migration of HUASMCs on different surfaces after 1 day of culture with donor supplement. Data are presented as the mean ± SD (*n* = 4) and analyzed using one‐way ANOVA, :*p* < 0.05, ::*p* < 0.01, :::*p* < 0.001.

Another key function of NO and endothelial glycocalyx molecules is the promotion of endothelialization at the vascular lesion sites and on the stent surfaces. Given this, the ECs behaviors on different substrates were also investigated. Similar to the result of SMCs adhesion and proliferation, ECs on the bare and aminated 316L SS did not show any difference regardless of whether the medium was supplemented with NO donor (**Figure** **7**A and Figure S6, Supporting Information). In contrast, the NO‐releasing groups, including the DOTA‐Cu, PAa/DOTA‐Cu, and HA@DOTA‐Cu surfaces, could efficiently enhance ECs adhesion and proliferation in the present of NO donor. Quantitative study revealed that, the dual‐functional HA@DOTA‐Cu surface provided the best surface microenvironment for ECs growth. The proliferation of ECs on HA@DOTA‐Cu surface was 1.6‐fold higher than that on the bare 316L SS surface after 72h of culture (Figure 7B). Further, the migration distances of ECs on different surfaces were examined. As clearly shown in Figure S7, Supporting Information, the migration of ECs on all groups showed no difference in culture medium without NO donor supplement. In contrast, in the present of donor supplement, all the NO‐releasing groups (i.e., DOTA‐Cu, PAa/DOTA‐Cu, and HA@DOTA‐Cu) showed observably enhanced cell migrations after 1‐day culture compared to the 316L SS group (Figure 7C). Although ECs migrations on the three NO‐releasing groups had no significant difference, the average migration distance and cell density on dual‐functional HA@DOTA‐Cu surface still was the longest, which were almost 2.2‐ and 1.4‐fold of those on the 316L SS surface, respectively (Figure 7D). According to the results, we could determine that the NO‐releasing ability and endothelium‐glycocalyx‐derived HA molecule would provide a biomimetic surface with ECs‐preferred properties.

**Figure 8:**
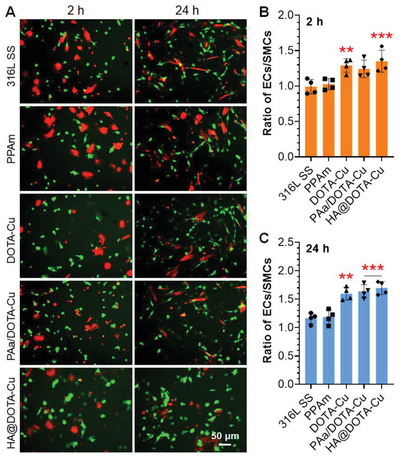
A) Green chloromethyl fluorescein diacetate (CMFDA) labeled HUVECs (green) and orange chloromethyl trimethyl rhodamine (CMTMR) labeled HUASMCs (orange) after 2 and 24 h coculture on the sample surfaces. B,C) The average ratios of adhered HUVECs and HUASMCs after 2 (B) and 24 h (C) of cell culture (*n* = 6). Data presented as mean ± SD and analyzed using a one‐way ANOVA, :*p* < 0.05, ::*p* < 0.01, :::*p* < 0.001.

ECs directly compete with SMCs in vivo, in particular, after vascular injury or stenting. Since the NO‐releasing property and HA molecule could provide EC‐friendly biological activity to enhance EC motility and inhibit excessive SMC growth, a competition experiment between ECs and SMCs was further carried out by co‐culturing HUVECs (green staining) and HUASMCs (red staining) (1:1) on different surfaces with NO donor supplement in the medium. As shown in **Figure** **8** and Figure S8, Supporting Information, the groups of DOTA‐Cu, PAa/DOTA‐Cu, and HA@DOTA‐Cu could elicit enhanced EC adhesion in the first 2 h. Over time, the adhesion and proliferation of ECs on all releasing groups exhibited a further enhancement against those of the SMCs after 24 h. In particular, the ratios of ECs to SMCs adhered on the dual‐functional group was the highest and nearly 1.5‐fold enhancement than those of the bare 316L SS surface, demonstrating their excellent selectivity for EC growth. In other word, this study justified the superiority of a dual‐functional surface for the inhibition SMC‐caused intimal hyperplasia and in situ generation of a pure endothelium in vivo.

### 2.6 In Vivo Cardiovascular Stent Implantation

The above experiments confirmed that the endothelium‐mimic dual‐functional surface was blood and vascular cell compatible. On one hand, the grafted glycocalyx molecules can provide a close range and positive regulation of surface microenvironment for vascular and blood cells; on the other hand, the in‐situ NO‐releasing property enables remote‐control of vascular homeostasis to reduce thrombogenesis, inhibit SMCs and promote ECs growth. The NO‐releasing and glycocalyx functions thus synergistically endow the surface with endothelium‐like properties. To further demonstrate the potential of this molecularly engineered stents for re‐endothelialization and prevention of ISR, long‐term implantation in the rabbit brachial artery was performed.

**Figure 9:**
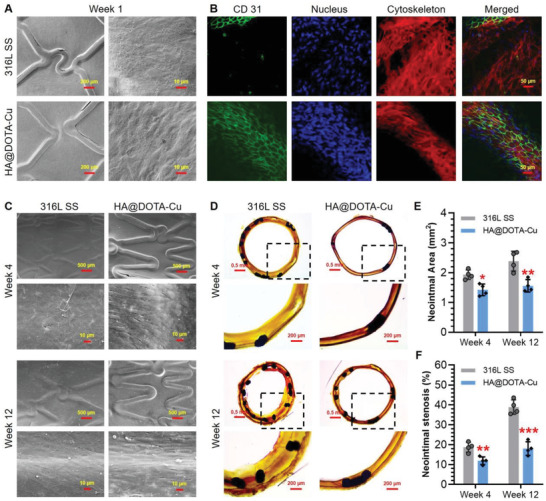
Long‐term stent implantation in vivo. A–C) Re‐endothelialization on the control and dual‐functional stents after implantation for 1, 4 and 12 weeks. D–F) Histomorphometric and quantitative analysis of ISR prevention in vivo. Data are presented as the mean ± SD of stent implantation experiments and analyzed using one‐way ANOVA, :*p* < 0.05, ::*p* < 0.01, :::*p* < 0.001.

The stented iliac arteries with implanted stents were harvested after 1, 4 and 12 weeks of stent deployment. To investigate early endothelialization, SEM and immunostaining assay were first used to observe the inner surface of stents at the first week. As clearly shown in the SEM images, the surface of bare 316L SS stent was fully covered by a thick layer of matrix, whereas the dual‐functional one (i.e., the HA@DOTA‐Cu‐grafted stents) still showed clear outline (**Figure** **9**A and Figure S9, Supporting Information). Immunostaining assay further revealed that the dual‐functional stents were well‐adhered with an intact layer of ECs (CD31 positive), while the heavily covered layer on the control stents was partly composed of ECs located just onto the edge of stent skeleton, probably due to the migration of ECs from the surrounding endothelial tissue (Figure 9B). This finding suggested the ECs‐friendly surface of the NO‐releasing andd HA‐grafted stents for early endothelialization in vivo. Given this, the long‐term re‐endothelialization on stents after 4 and 12 weeks of implantation was further evaluated. As shown in Figure 9C, although the surface of bare 316L SS stent could be covered by a layer of cells at week 4 and 12, most of them were inconsistent with the spindle morphology of ECs. In contrast, the HA@DOTA‐Cu‐grafted vascular stent was fully covered by an intact monolayer of ECs, which showed elongated morphology that were oriented to the blood flow direction due to shear stress. These results confirmed that the dual‐functional surface with optimized NO‐generation and glycocalyx functions could significantly promote re‐endothelialization process on vascular stents in vivo.

Finally, histomorphometric analysis of the stent cross sections was performed to determine the practical effects on prevention of intimal hyperplasia and restenosis. After hematoxylin eosin (HE) staining, typical tissue slicing of the arteries with implanted stents were presented in Figure 9D. As compared to the control, the dual‐functional vascular stent showed remarkably reduced neointimal hyperplasia during the implantation period. In particular, at the week 12, the dual‐functional stents showed significant decrease of the neointimal stenosis ratio (Figure 9E, 34.4% vs 20.4%) and mean neointimal area (Figure 9F, 2.43 mm^2^ vs 1.53 mm^2^) as compared with the controls. Undoubtedly, the endothelium‐mimic NO‐releasing and HA‐grafted vascular stents could significantly reduce ISR in vivo.

## 3 Conclusion

In summary, we developed a novel layer‐by‐layer grafting strategy for the fabrication of endothelium‐like surface with dual functions on the cardiovascular scaffolds. To mimic the endothelial functions capable of remote and close‐range regulation of vascular microenvironment, a NO‐generating compound DOTA‐Cu^2+^ and an endothelial polysaccharide matrix molecule HA were sequentially immobilized on the surface of vascular stents through aqueous amidation. The synergy of NO‐releasing and endothelial glycocalyx functions at the interface of stents led to efficient antithrombosis, smooth muscle cell (SMC) inhibition, and perfect endothelial cell (EC)‐compatibility in vitro. Moreover, the endothelium‐mimic dual‐functional stents also showed efficient antithrombogenesis ex vivo, rapid endothelialization, and long‐term prevention of restenosis in vivo. In a word, the study in this work might provide new solutions for not only multicomponent surface biofunctionalization but also a highly bionic surface bioengineering strategy for vascular scaffolds with endothelium‐like functions, which would contribute greatly to the improvement of clinical outcomes.

## 4 Experimental Section

##### Materials

DOTA was purchased from Shanghai Aladdin Bio‐Chem Technology Co., Ltd. PAa, HA (molecular weight: 1–2 × 10^5^ Da), CuCl_2_·2H_2_O, Tris‐HCl, GSNO, GSH, cGMP Enzyme Immunoassay Kit, glutaraldehyde, MES, NHS, and EDC were purchased from Sigma‐Aldrich. DMEM‐F12 medium and fetal bovine serum (FBS) were from GE Healthcare Life Sciences. Cell Tracker Green CMFDA and Orange CMTMR were bought from Thermo Fisher Scientific. CCK‐8 was purchased from Dojindo.

##### Preparation of PPAm Coating

The process of PPAm coating preparation is described in detail elsewhere.^[^ ^21^ ^]^ Briefly, the PPAm coating was deposited onto mirror polished 316L SS using capacitative plasma excited by an external coil with a 13.56 MHz pulsed radio frequency (RF) excitation. The allylamine (Sigma, Purity ≥ 99.0%) vapor was used as precursor gas.^[^ ^22^ ^]^ The 316L SS substrates were argon plasma sputtered for 5 min prior to the deposition of the PPAm coating. Then, the PPAm coating was fabricated under 15 Pa system pressure, 4.5 sccm Ar, 80 V negative bias voltage, 30 W RF power, and 40% of pulsed duty cycle (*t*_on_ = 20 ms, *t*_off =_ 30 ms). After 45 min of deposition, the PPAm‐coated 316L SS was thermally treated at 150 °C under 1.5 × 10^−4^ Pa.^[^ ^16^, ^22^ ^]^

##### Sequential Co‐Immobilization of DOTA‐Cu^2+^ and HA

DOTA‐Cu^2+^ complex and HA were sequentially immobilized on PPAm coating by covalent immobilization. First, DOTA and CuCl_2_·2H_2_O equivalent molar quantity were mixed and dissolved by deionized water. After reaction for 30 min under ultrasound, the precipitation of DOTA‐Cu^2+^ complex was obtained by centrifugation at 10 000 rpm. After 9 cycles of washing by distilled water and centrifugation the pure DOTA‐Cu^2+^ complex was obtained. Then, the complex of DOTA‐Cu^2+^ (1 mg mL^−1^) were activated by WSC system (EDC (1 mg mL^−1^) and NHS (0.24 mg mL^−1^) in a MES (9.76 mg mL^−1^) buffer with the pH adjusted to 5.4 by NaOH). The PPAm‐coated 316L SS substrates and cardiovascular stents were subsequently immersed the above solution of DOTA‐Cu^2+^. After grafting for 12 h, the samples grafted by DOTA‐Cu^2+^ were washed by PBS and distilled water. The PPAm‐coated sample immobilized with DOTA‐Cu^2+^ was marked as DOTA‐Cu. To get high HA grafting density, PAa was thereby employed to amplify the reaction sites by transferring the residual carboxyl groups of DOTA‐Cu into multiple amino groups. The samples of DOTA‐Cu were activated WSC system for 30 min, and then 0.1 mg mL^−1^ PAa. After grafting for 12 h, the PAa‐grafted samples (PAa/DOTA‐Cu) were washed with PBS and distilled water in sequence (15 min × 3 times). Finally, HA (2 mg mL^−1^) with molecular weight ranging from 100 to 200 kda was immobilized on the surface of PAa/DOTA‐Cu under the activation of WSC solution system. After 12 h reaction, the specimens were rinsed with PBS and distilled water, respectively, and marked as HA@DOTA‐Cu.

QCM‐D (Q‐sense AB, Sweden) was used to quantify the mass of the molecules bound to the prefabricated coatings. First, the AT‐cut 5 MHz Au coated quartz crystal (diameter of the Au film: 10 mm) was functionalized by a PPAm film with a thickness of 10 nm approximately. Then, the PPAm‐coated quartz crystal was mounted in the QCM‐D chamber, then MES (pH 5.4) solution was injected continuously at a rate of 50 µL min^−1^ until the QCM baseline stabilized. After that, 1 mg mL^−1^ of DOTA‐Cu^2+^ dissolved by WSC solution was injected until the curve equilibrated. Afterward, PBS (pH 7.4) was perfused to remove the unbound DOTA‐Cu^2+^. The final mass (∆*m*) of the grafted‐molecule was calculated based on Sauerbrey equation,^[^ ^23^ ^]^ the frequency shift (∆ƒ) of the quartz crystal was converted into mass change (∆*m*) on the electrode surface and calculated through the Sauerbrey equation. This method is also used for measuring the grafted mass of both PAa and HA.

##### Characterization of the Coatings

The surface chemical compositions of the specimens were investigated by XPS (K‐Alpha, Thermo Electron, USA). The instrument was equipped with a monochromatic Al K*α* (1486.6 eV) X‐ray source operated at 12 kV × 15 mA, and a pressure of 3 × 10^−7^ Pa. The graphitic carbon peak (284.8 eV) was used as a reference for charge correction. GATR‐FTIR measurement was performed to analyze the chemical structure of the substrates using (Nicolet MODEL 5700). The WCA was measured by Krüss GmbH DSA 100 Mk 2 goniometer (Hamburg, Germany) at room temperature.

##### Catalytic Release Rate of NO

Real‐time catalytic release of NO from specimens was detected by chemiluminescence NO analyzer (NOA, Seivers 280i, Boulder, CO). In detail, 5 mL of testing PBS solution consisting of 10 µm GSNO and 10 µm GSH, was previously added to the reaction chamber. After 5 min of baseline calibration of NO level, a 316L SS foil (0.5 cm × 1 cm) functionalized with the HA@DOTA‐Cu coating was plunged into the reaction chamber. N_2_ gas with controllable airflow size delivers RSNO decomposited NO to the NO analyzer. The amount of NO generated by the HA@DOTA‐Cu coating was calculated based on the calibration curves of the NOA, as detailed in literature. For the evaluation of the long‐term catalytic stability of the HA@DOTA‐Cu‐coated surface, the HA@DOTA‐Cu‐coated substrates were in advance immersed in PBS solution for 1, 5, 10, 15, 20, and 30 days, respectively, the PBS solution was replaced every 5 days.

##### In Vitro Test of Platelet Adhesion

The fresh human platelet rich plasma (PRP) obtained from the central blood station of Chengdu, China, was used in this study following ethics standards. Considering the lack of endogenous RSNO preserved in PRP due to poor chemical stability of RSNO, moderate amount of RSNO consisting of 10 µm GSNO and 10 µm GSH was supplemented in PRP for the in vitro platelet test. First, 200 µL of PRP was dropped on each sample and incubated for 30 min, at 37 °C. Next, the samples were washed three times with PBS and fixed with 2.5% glutaraldehyde overnight, and then dehydrated and dealcoholized.^[^ ^22^ ^]^ Finally, the samples were examined by SEM.

cGMP expression of platelets adhered on samples was analyzed using cGMP ELISA kit. Similar to the platelet adhesion experiment, the samples were first cultured with 1 mL of PRP supplemented with 10 µm GSNO and 10 µm GSH for 30 min. Then, 100 µL of Triton X (10%) was added into each sample and treated with ultrasonic (5min). Finally, the supernatant was centrifuged and collected at 2500 r.p.m. and then analyzed following the instruction of the cGMP ELISA kit.^[^ ^23^ ^]^

##### Cell Culture

HUVECs were isolated from human umbilical cord by enzyme digestion. The study protocol complies with the principles of the Declaration of Helsinki and was approved by the Ethics Committee of Southwest University Jiaotong University. HUASMCs were obtained from human umbilical artery by small tissue culture method. Both cells were cultured in DMEM‐F12 supplemented with 20% FBS in a humidified incubator containing 5% CO_2_ at 37 °C.

##### Adhesion and Growth of HUASMCs and HUVECs

In order to evaluate the effects of the HA@DOTA‐Cu coating on growth behavior of HUASMCs and HUVECs, cell culture media with or without donor supplements (10 µm GSNO, 10 µm GSH, NO donor was supplemented every 6 h) were used in this study. Cells were seeded on the surfaces of the samples with a density of 2 × 10^4^ cells mL^−1^ for 2 h, 24 h, and 72 h, respectively. After that, cells grown on samples were fixed with glutaraldehyde (2.5%) and stained with fluorescence to evaluate the morphology of the cells. CCK‐8 was used to evaluate proliferation of cells after culture of 24 and 72 h.

##### Migration of HUASMCs and HUVECs

In this study, L‐shape 316L SS (0.8 cm × 2 cm) foil was used for the cell migration assays. In brief, half of the L‐shape 316L SS (0.8 cm × 2 cm) foil was functionalized by the coating, and the other half of the bare 316L SS was used for growing a compact cell monolayer through culture of HUASMCs and HUVECs at a density of 5 × 10^5^ cells mL^−1^ for 6 h. After that, the sample was turned over, which makes the coated‐half parallel to the bottom of the 24‐well plate, then the cells from compact cell monolayer started to migrate from the bare side to coated‐side. During the incubation, NO donor was supplemented in culture medium every 4 h. Finally, after 24 h of incubation, the sample was washed by PBS, fixed by glutaraldehyde (2.5%) and stained with Rhodamine 123. The migrated cells on samples were inspected using Leica DMRX fluorescence microscope.

##### Co‐Culture of HUASMCs and HUVECs

First, HUASMCs were prelabeled with orange chloromethyl trimethyl rhodamine (CMTMR), while HUVECs were prelabeled with green chloromethyl fluorescein diacetate (CMFDA) according to product instructions.^[^ ^24^ ^]^ Then, two types of fluorescent labeled cells were digested with trypsin (0.25% wt) and mixed at a ratio of 1:1 using DMEM‐F12 medium supplemented with 10% FBS. The densities of two kinds of cells were 2 × 10^4^ cells mL^−1^. The suspensions of mixed cells were subsequently added to the 24‐well plate containing the samples and cocultured for 2 h and 24 h, respectively, in a cell incubator at 37 °C in damp air with 5% CO_2_. The cells grown on the sample were finally evaluated and photographed using OLYMPUS fluorescence microscope. The amounts of the adherent cells were calculated from more than 12 images by Image J.

##### Ex Vivo Thrombogenicity Test

All procedures for animal experiments were approved by the Animal Care and Use Committee of Southwest Jiaotong University and complied with the Guide for the Care and Use of Laboratory Animal of the National Institutes of Health . Six healthy New Zealand white rabbits (2.5–3.5 kg) were used for ex vivo thrombogenicity test. After general anesthesia by 3% pentobarbital sodium salt (1mg kg^−1^), the left carotid artery and the right jugular vein of the rabbit were separated and then connected with a commercially available polyvinyl chloride (PVC) cardiopulmonary perfusion tubing. The uncoated, and PPAm‐, DOTA‐Cu‐, PAa/DOTA‐Cu‐, and HA@DOTA‐Cu‐coated 316L SS foils (1.5 cm × 0.85 cm) were curled and installed inside the inner walls of PVC tubes. 10 µm GSNO and 10 µm GSH were supplemented during the experiment. After 2 h of blood circulation, the samples were removed from the circulation system with a partial tubing containing the testing samples and rinsed with PBS (pH 7.4). The cross section of the testing tubing containing the sample was photographed to analyze the occlusion rate. The occlusion rate was calculated by measuring the cross‐sectional area of the lumen before and after circulation. The blood flow rate at the end of circulation was normalized to that before the start of circulation under the same pressure pump condition. After that, the tested sample was taken from the tubing for photographing and weighting. Finally, the sample was fixed with glutaraldehyde (2.5% in physiological saline) for SEM observation.

##### In Vivo Stent Implantation

Twelve of adult New Zealand white rabbits with weight ranging from 3.0 to 3.5 kg were used for stent implantation. Rabbits were fed with high‐fat diet for two weeks before stent implantation. After general anesthesia, the uncoated and HA@DOTA‐Cu‐coated 316L SS stents were implanted into bilaterally iliac arteries of rabbits, respectively, and anti‐inflammatory therapy using penicillin and systemic anticoagulant therapy using warfarin lasted for a week after stent implantation. The rabbits were euthanized after implantation at 1 week, 1 month, and 3 months, respectively, and the stented arteries were harvested and gently washed with heparinized saline. In the case of groups of 1 week, the stented artery was opened longitudinally and fixed with polyformaldehyde (4% in PBS) for 12 h, then was immersed in a solution of mouse antirabbit CD31 antibody (Novus Biologicals, 1:300 dilution in PBS) for 2 h. After that, it was subsequently immersed into Alexa Fluor® 488‐conjugated goat antimouse IgG secondary antibody (Absin Bioscience, 1:800 dilution in PBS) for another 2 h, followed by staining with 4’,6‐diamidino‐2‐phenylindole (DAPI) (1 µm) and FITC‐Phalloidin (100 nm) for 30 min, respectively. Finally, it was washed by PBS for three times and clamped within two piece of glass slide to make the stented artery flat. It was observed by laser scanning confocal microscopy (LSCM, Nikon A1plus, Japan). The cell nuclei stained with DAPI shows blue (excitation wavelength: 405 nm), the cell membrane labeled by CD31 antibody exhibits green (excitation wavelength: 450 nm) and the cytoskeleton binding with FITC‐Phalloidin presents red (excitation wavelength: 515 nm). For the groups of 1 and months, the stented artery was transversely cut into two pieces, one of them was fixed with polyformaldehyde (10%) for 48 h, and then was embedded in the resin, and subjected to hard tissue section for histological evaluation. The other half was cut lengthwise, flattened and fixed with glutaraldehyde (2.5% in physiological saline) for 48 h to observe the surface morphology by SEM.

##### Statistical Analysis

All data are presented as mean ± standard deviation. Statistical analysis was carried out using SPSS software, employing a one‐way ANOVA as detailed in the figure captions. Tests that had an alpha level for significance set at *p* < 0.05 were considered significantly different.

## Conflict of Interest

The authors declare no conflict of interest.

## Supporting information

Supporting Information
