# Supplementary material for: Mimicking the Nitric Oxide‐Releasing and Glycocalyx Functions of Endothelium on Vascular Stent Surfaces
Source: Adv Sci (Weinh). 2020 Sep 27;7(21):2002330. doi: 10.1002/advs.202002330 (PMC7610264; doi:10.1002/advs.202002330)
Supplement: Supplementary file 1 — Supporting Information [file ADVS-7-2002330-s001.pdf]

© 2020 Wiley-VCH GmbH

Supporting Information

for *Adv. Sci.*, DOI: 10.1002/advs.202002330

Mimicking the Nitric Oxide-Releasing and Glycocalyx Functions of Endothelium on Vascular Stent Surfaces

*Nan Lyu, Zeyu Du, Hua Qiu, Peng Gao, Qin Yao, Kaiqin Xiong, Qiufen Tu, Xiangyang Li, Binghai Chen, Miao Wang, Guoqing Pan,**
Nan Huang, and Zhilu Yang**

© 2020 Wiley-VCH GmbH

Supporting Information

Mimicking the Nitric Oxide-Releasing and Glycocalyx Functions of Endothelium on Vascular Stent Surfaces

Nan Lyu, Zeyu Du, Hua Qiu, Peng Gao, Qin Yao, Kaiqin Xiong, Qiufen Tu, Xiangyang Li, Miao Wang, Binghai Chen, Guoqing Pan, Nan Huang,* & Zhilu Yang**

N Lyu, Z. Du, H. Qiu, P. Gao, Dr. K. Xiong, Prof. Dr. Q. Tu, Dr. X. Li, Prof. Dr. N. Huang, Prof. Dr. Z. Yang
Key Lab of Advanced Technology of Materials of Education Ministry, School of Materials Science and Engineering, Southwest Jiaotong University, Chengdu, 610031, China.
E-mail: huangnan1956@163.com, zhiluyang1029@swjtu.edu.cn

Q. Yao, Prof. Dr. B. Chen
Department of Urology, Affiliated Hospital of Jiangsu University, Zhenjiang, Jiangsu, China

Dr. M. Wang, Prof. Dr. G. Pan
Institute for Advanced Materials, School of Materials Science and Engineering, Jiangsu University, Zhenjiang 212013, Jiangsu, China
E-mail addresses: panguoqing@ujs.eu.cn

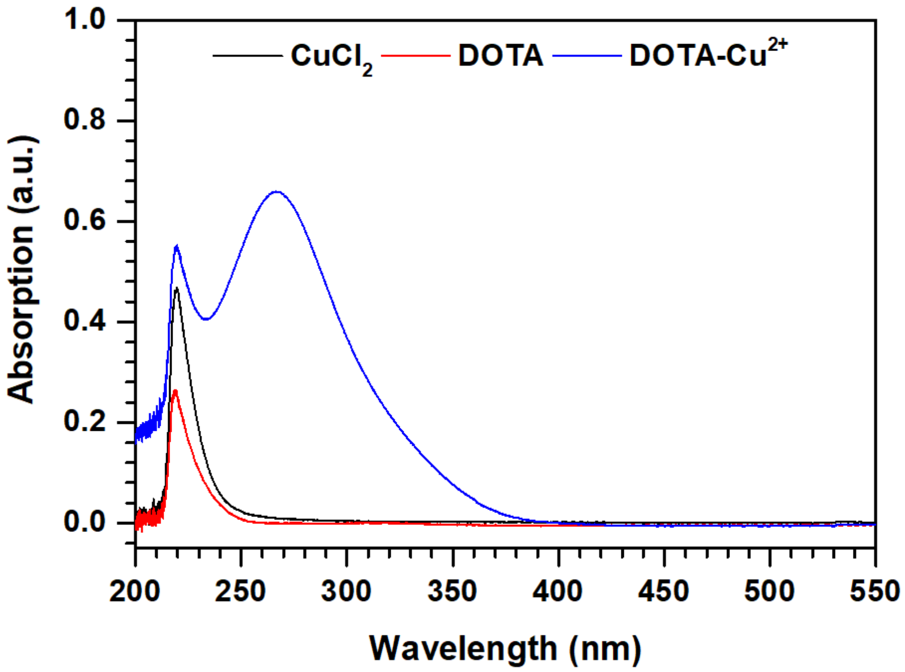

Figure S1. *Testing of copper chloride solution, DOTA solution and DOTA-Cu^{2+} solution by UV spectrophotometer. The analysis results found that the copper chloride solution and the DOTA solution only had a single absorption peak at the UV wavelength of 219 nm. The DOTA-Cu^{2+} solution not only has an absorption peak at an ultraviolet wavelength of 219 nm, but also an absorption peak at a wavelength of 266 nm. This newly generated absorption peak should be due to the chelation of copper ions by DOTA. Therefore, it has been proved by UV spectrophotometer experiments that copper ions can be successfully chelated in DOTA.*

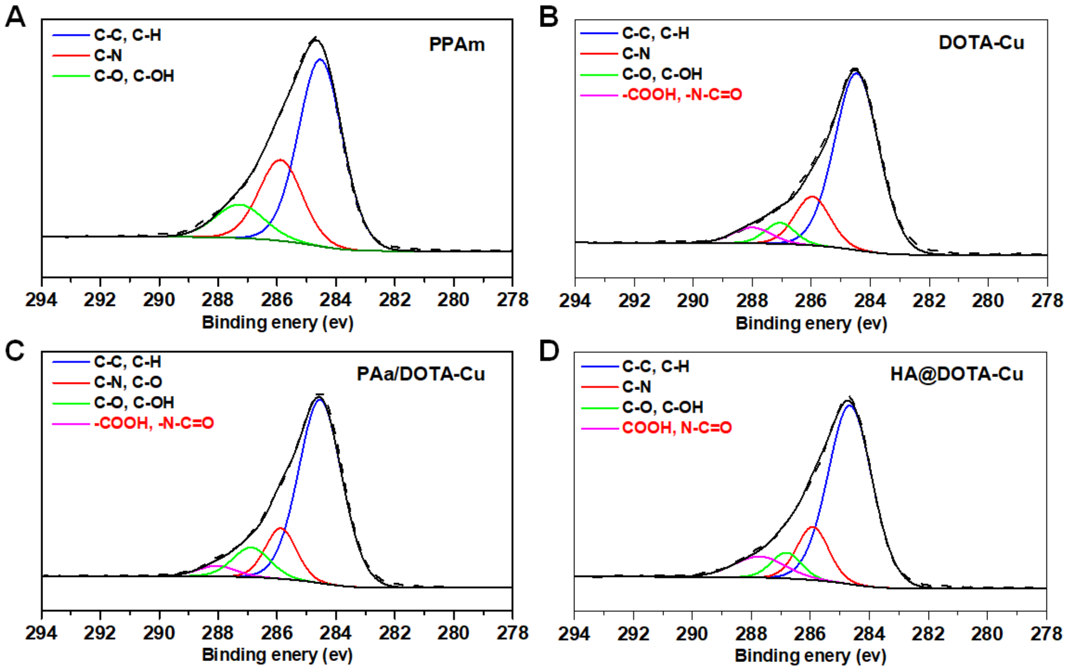

Figure S2. *High resolution spectra of A) C1s signal on the 316L SS, PPAm, DOTA-Cu, PAa/DOTA-Cu and HA@DOTA-Cu surfaces.*

The C 1s high resolution XPS spectra of 316L SS, PPAm, DOTA-Cu, PAa/DOTA-Cu and HA@DOTA-Cu were generated. The C1s high-resolution spectra revealed at least four overlapped peaks. The peak at 284.8 eV was assigned to C-C and C-H bonds, which was used as a reference point to determine that all the spectra had shifted. A second peak at 285.7 eV corresponds to C-N bond. The third component of C 1s was shifted by approximately 2.0 eV, and was assigned mainly to C-O, C-N and C≡N groups. A fourth peak at 287.9 eV possibly corresponds to C=O and N–C=O bonds. After DOTA-Cu^{II} immobilization, the further change that was observed was a shift of the peak at 288.4 eV, most likely due to the introduction of carboxyl groups (–COOH) and N–C=O which formed in immobilized. After HA immobilization, XPS spectra also changed significantly, most likely due to the introduction of carboxyl groups (–COOH) and N–C=O which formed in immobilized.

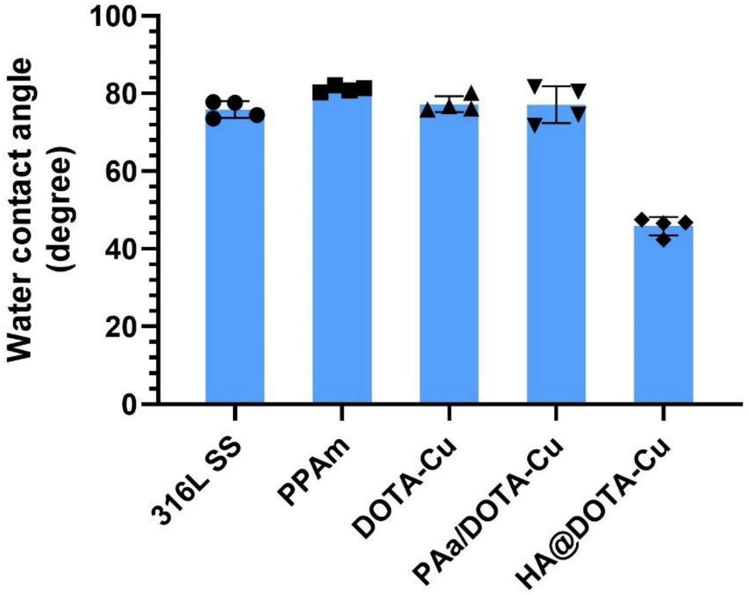

Figure S3. *The water contact angles of the 316L SS, PPAm, DOTA-Cu, PAa/DOTA-Cu and HA@DOTA-Cu surfaces.*

The water contact angle of 316L SS and PPAm was 55.9±4.2◦ and 81.2±0.8◦ respectively. The water contact angle of 316L SS increased significantly, which indicated that the plasma polyallylamine film was successfully deposited on 316L SS surface. The water contact angles of the DOTA-Cu and PAa/DOTA-Cu surfaces were 77.2 ± 2.0◦ and 77.1 ± 4.8◦, respectively. The water contact angle of the HA@DOTA-Cu sample was 45.9 ± 2.4◦. The large decrease in the water contact angle of the sample indicates a significant increase in hydrophilicity, which is due to the successful grafting of hyaluronic acid.

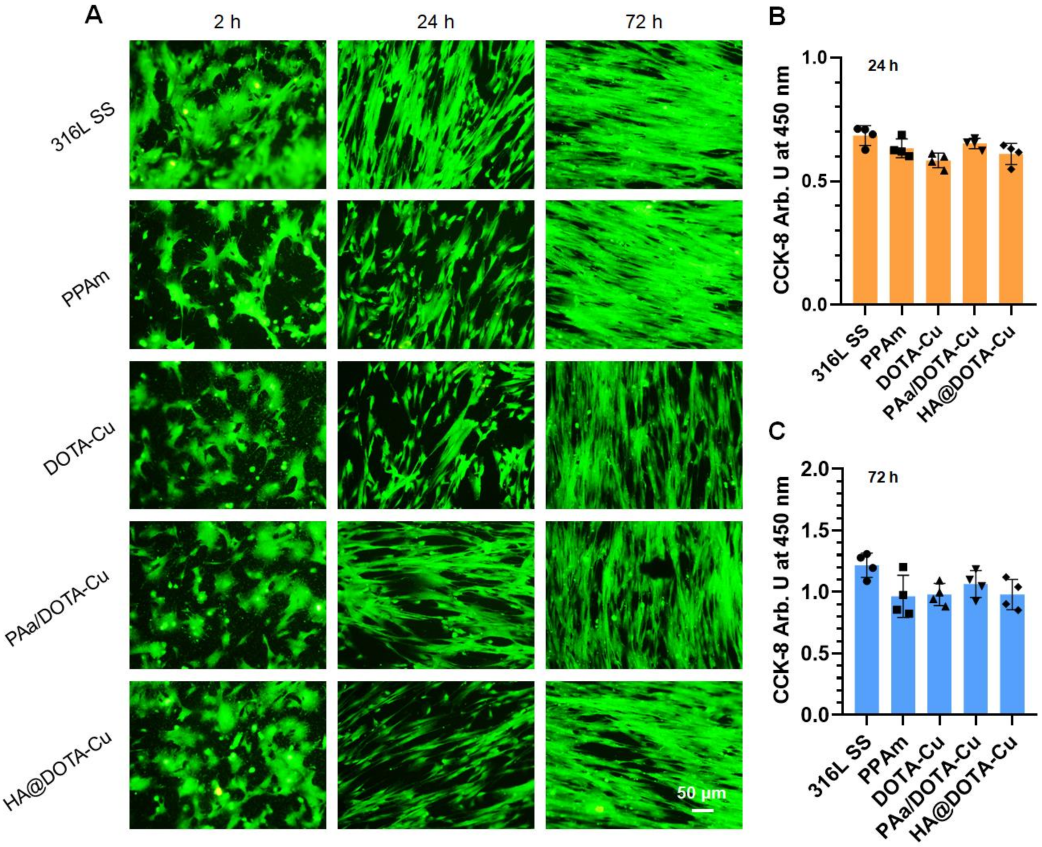

Figure S4. *A) Fluorescence staining of HUASMCs on samples surfaces after culture for 2, 24 and 72 h without NO donor. Proliferation of HUASMCs cultured in cell media with NO donor for 24 B) and 72 h. C) Data are presented as the mean ± SD (n = 4) and analyzed using one-way ANOVA, *p < 0.05, **p < 0.01, ***p < 0.001*

In the non-donor environment, it was found from the fluorescence staining map that there was no significant difference in the number of HUASMCs adhering to the surface of each group of the culture time of 2 hours. There was no significant difference in proliferation between HUASMCs after 24 hours and 72 hours of incubation on various samples.

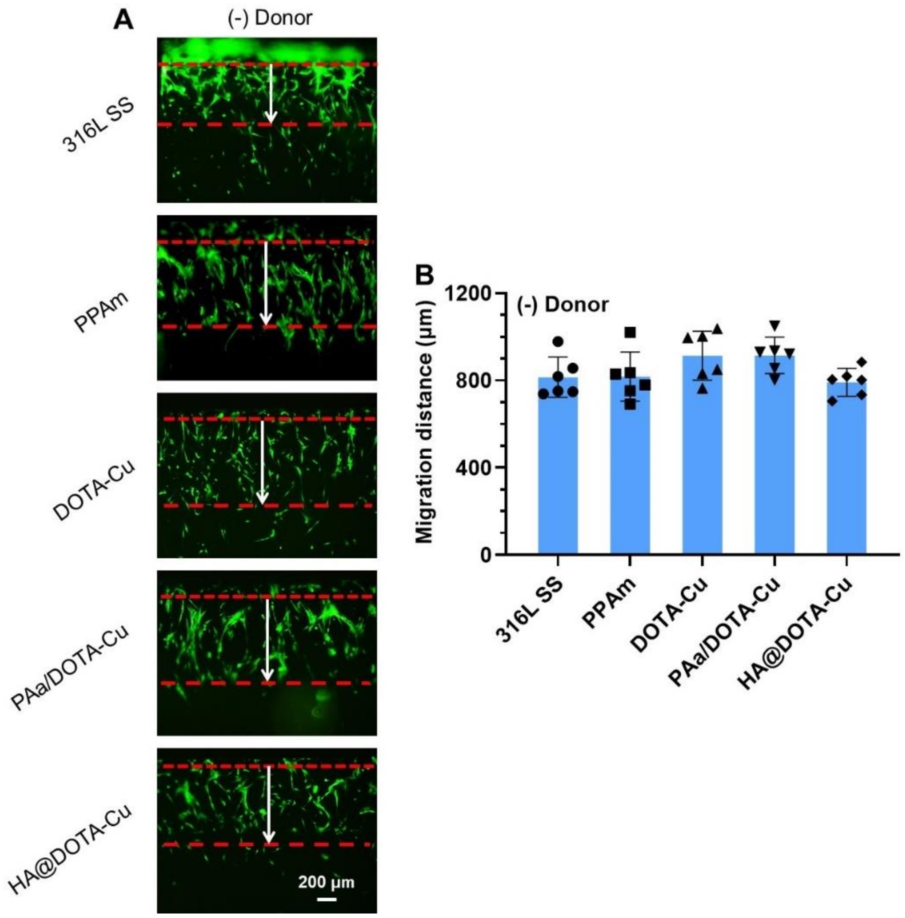

Figure S5. *Migration A) of HUASMCs on 316L SS, PPAm, DOTA-Cu, PAa/DOTA-Cu and HA@DOTA-Cu surfaces. The migration distance B) was calculated from at least 12 images.*

Smooth muscle cell migration statistics showed that there was no statistical difference in the distance of migration in the samples without donor addition. The smooth muscle cells on the surface of all samples migrated at a distance of 800-900 μm for 24 hours.

In the donor group, the smooth muscle migration distance of the 316L SS sample was 656 ±148 μm, and the smooth muscle migration distance of the PPAm surface was 770 ± 95 μm micrometers. On the surface of the sample with NO catalytic release, the smooth muscle migration was significantly reduced. The migration distance of the DOTA-Cu surface was only 524 ± 108 μm, which was 364 μm lower than that of the non-donor sample. The surface migration distance of HA@DOTA-Cu is 276 μm, which is 516 μm lower than that of non-donor group, and 186% lower than that of non-donor group. It is indicated that the sample of HA@DOTA-Cu can sufficiently inhibit the migration of smooth muscle.

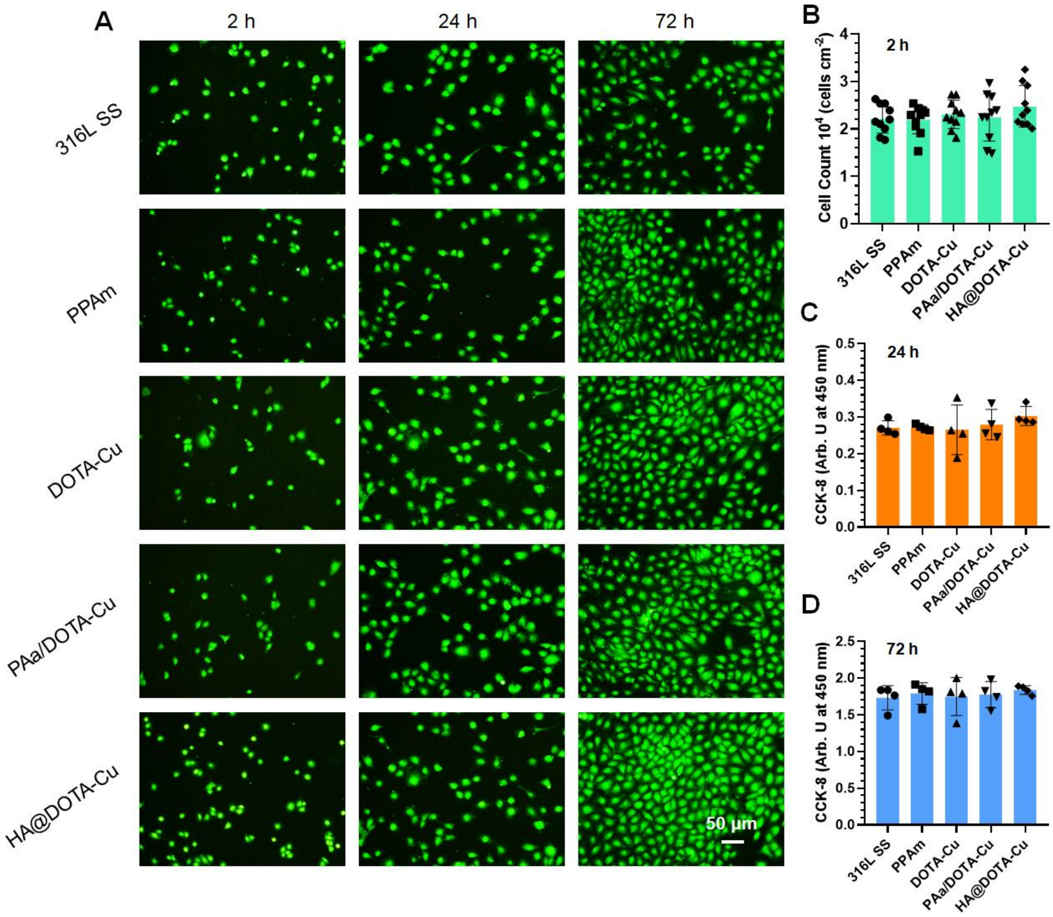

Figure S6. *A) Fluorescence staining of HUVECs on the samples surfaces after culture for 2, 24 and 72 h without NO donor. B) Cell count (calculated from at least 12 images), and Proliferation of HUVECs cultured in cell media with NO donor for 24 and 72 h C). Data are presented as the mean ± SD (n = 4) and analyzed using one-way ANOVA, *p < 0.05, **p < 0.01, ***p < 0.001.*

In the non-donor environment, it was found from the fluorescence staining map that there was no significant difference in the number of HUVECs adhering to the surface of each group of the culture time of 2 hours. There was no significant difference in proliferation between HUVECs after 24 hours and 72 hours of incubation on various samples.

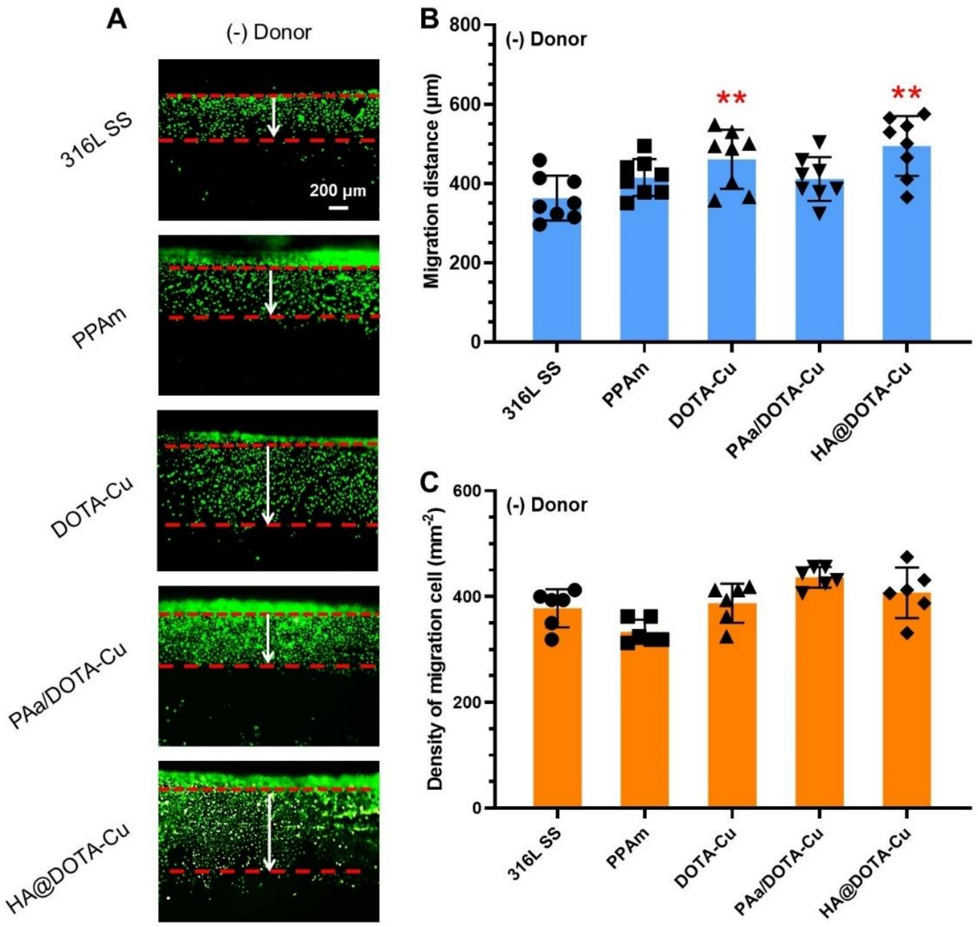

Figure S7. *Migration A) of HUVECs on 316L SS, PPAm, DOTA-Cu, PAa/DOTA-Cu and HA@DOTA-Cu after one day cultrure in medium without NO donor. The migration distance B) and cell density C) after migration was calculated from at least 12 images.*

In the 24 hours culture of the non-donor group, the migration distance of HUVECs on different sample surfaces did not show obvious regularity. The sample group with NO catalyzed release in the donor group showed a significant improvement compared to the 316L SS surface. Specifically, the migration distance of the HA@DOTA-Cu sample relative to the surface of the 316L SS was increased by 453 μm, and the increase rate was 226%. The density of HUVECs after migration in the non-donor group did not differ significantly among the samples in each group. In donors, the HUVECs density of three groups of samples with NO catalytic release was higher than 316L SS and PPAm groups. The HUVECs density of the 316L SS sample group was 377 (cell/mm^2), compared to 650 (cell/mm^{-2}) for the HA@DOTA-Cu sample group. The results show that NO catalytic release is beneficial to endothelial cell migration and can accelerate the completion of endothelialization.

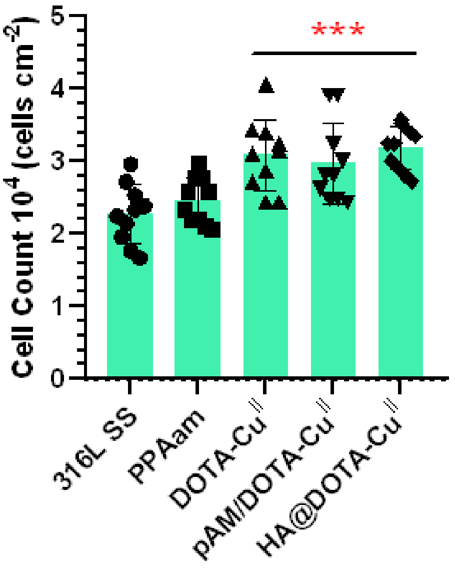

Figure S8. *A) Fluorescence staining of HUVECs on samples surfaces after culture for 2 with NO donor.*

In the NO donor environment, fluorescence staining showed that there were significant differences in the number of HUVECs adhered to the cell surface after 2 hours of culture. The results showed that the number of endothelial cells in the sample group with no catalytic release was significantly higher than that in the sample without catalytic release of nitric oxide.

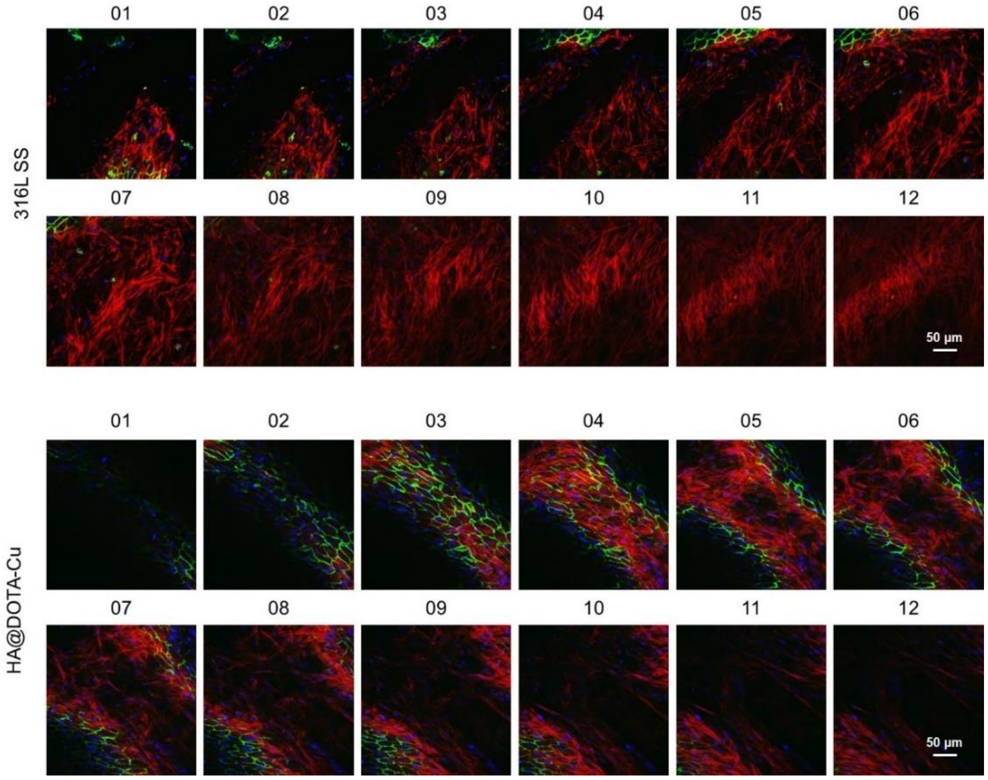

Figure S9. *Results of immunostaining assay at different focal length depths, CD31 was labeled by phycoerythrin, cell nuclei were stained by DAPI, Cytoskeleton were stained by FITC-Phalloidin.*

LSCM observation started from the inner surface of the blood vessel containing the stent rib. The first photo was named as 01, and then the focus was taken from inside to outside at every 1.5 micron and the photograph was taken until below the intimal layer. A comprehensive observation showed that there were a large number of nuclei and cytoskeleton on the surface of the 316L SS stent sample, but there was no green fluorescence signal of CD31, indicating that the surface of the bare metal stent was not covered by endothelial cells. However, there were a large number of green fluorescent signals on CD31 on both sides of the stent, indicating that the endothelial cells around the scaffold did not migrate to the scaffold after one week of stent implantation, further showing the poor cell compatibility of bare metal stent. In HA@DOTA-Cu coated stents, a large number of nuclei and cytoskeletons were found on the surface of stents, while a large number of endothelial cells were also found on the surface of stents. It is indicated that after one week of stent implantation, part of the endothelial cells had migrated from the endothelial layer on both sides of the stent to the surface of the stent. Rapid endothelialization is essential for the formation of late thrombosis and restenosis with inhibition of cardiovascular stent implantation, so HA@DOTA-Cu coated stents have better biocompatibility than bare metal stents.
